# Supplementary material for: MetaQTL: a package of new computational methods for the meta-analysis of QTL mapping experiments
Source: BMC Bioinformatics. 2007 Feb 8;8:49. doi: 10.1186/1471-2105-8-49 (PMC1808479; doi:10.1186/1471-2105-8-49)
Supplement: Additional File 7 — MetaQTL Package : jar file and tutorial. This Zip archive contains both the MetaQTL JAR file and the files of the tutorial. [file 1471-2105-8-49-S7.zip › org.inra.metaqtl/doc/org/thalia/bio/entity/bean/OntologyBean.html]

OntologyBean


|  |  |  |
| --- | --- | --- |
| **Overview** \| **Package** \| **Class** \| **Use** \| **Tree** \| **Deprecated** \| **Index** \| **Help** | |  |
| **PREV CLASS**   **NEXT CLASS** | **FRAMES**    **NO FRAMES**     **All Classes** |  |
| SUMMARY: NESTED \| FIELD \| CONSTR \| METHOD | DETAIL: FIELD \| CONSTR \| METHOD |  |


---


## org.thalia.bio.entity.bean Class OntologyBean

```
java.lang.Object
  org.thalia.bio.entity.bean.BioEntityBean
      org.thalia.bio.entity.bean.OntologyBean
```

---

``` public class OntologyBean extends BioEntityBean ```

---

| **Field Summary** | |
| --- | --- |
| `java.lang.String` | `function`             The function of the ontology. |
| `OntologyTermBean` | `root`             The root of the ontology. |

**Fields inherited from class org.thalia.bio.entity.bean.BioEntityBean**

`name, properties`


**Constructor Summary**

`OntologyBean()`


**Method Summary**

**Methods inherited from class org.thalia.bio.entity.bean.BioEntityBean**

`getName, getProperties, setName, setProperties`

**Methods inherited from class java.lang.Object**

`clone, equals, finalize, getClass, hashCode, notify, notifyAll, toString, wait, wait, wait`

**Field Detail**

### function

```
public java.lang.String function
```

:   The function of the ontology.

---


### root

```
public OntologyTermBean root
```

:   The root of the ontology.


**Constructor Detail**

### OntologyBean

```
public OntologyBean()
```


---


|  |  |  |
| --- | --- | --- |
| **Overview** \| **Package** \| **Class** \| **Use** \| **Tree** \| **Deprecated** \| **Index** \| **Help** | |  |
| **PREV CLASS**   **NEXT CLASS** | **FRAMES**    **NO FRAMES**     **All Classes** |  |
| SUMMARY: NESTED \| FIELD \| CONSTR \| METHOD | DETAIL: FIELD \| CONSTR \| METHOD |  |


---
